# A Novel Intergenic ETnII-β Insertion Mutation Causes Multiple Malformations in *Polypodia* Mice

**DOI:** 10.1371/journal.pgen.1003967

**Published:** 2013-12-05

**Authors:** Jessica A. Lehoczky, Peedikayil E. Thomas, Kevin M. Patrie, Kailey M. Owens, Lisa M. Villarreal, Kenneth Galbraith, Joe Washburn, Craig N. Johnson, Bryant Gavino, Alexander D. Borowsky, Kathleen J. Millen, Paul Wakenight, William Law, Margaret L. Van Keuren, Galina Gavrilina, Elizabeth D. Hughes, Thomas L. Saunders, Lesil Brihn, Joseph H. Nadeau, Jeffrey W. Innis

**Affiliations:** 1 Department of Human Genetics, University of Michigan, Ann Arbor, Michigan, United States of America; 2 Pediatrics, University of Michigan, Ann Arbor, Michigan, United States of America; 3 Biomedical Research Core Facilities, DNA Sequencing Core Lab, University of Michigan, Ann Arbor, Michigan, United States of America; 4 Murine Molecular Constructs Laboratory-MMCL Mouse Biology Program, University of California, Davis, California, United States of America; 5 University of California, Davis, Center for Comparative Medicine and Comprehensive Cancer Center, Department of Pathology and Laboratory Medicine, Davis, California, United States of America; 6 Division of Genetic Medicine, Department of Pediatrics, Seattle Children's Hospital, Seattle, Washington, United States of America; 7 Transgenic Animal Model Core Lab, University of Michigan, Ann Arbor, Michigan, United States of America; 8 Department of Genetics, Case Western Reserve University, Cleveland, Ohio, United States of America; 9 Pacific Northwest Research Institute, Seattle, Washington, United States of America; Stanford University School of Medicine, United States of America

## Abstract

Mouse early transposon insertions are responsible for ∼10% of spontaneous mutant phenotypes. We previously reported the phenotypes and genetic mapping of *Polypodia*, (*Ppd*), a spontaneous, X-linked dominant mutation with profound effects on body plan morphogenesis. Our new data shows that mutant mice are not born in expected Mendelian ratios secondary to loss after E9.5. In addition, we refined the *Ppd* genetic interval and discovered a novel ETnII-β early transposon insertion between the genes for *Dusp9* and *Pnck*. The ETn inserted 1.6 kb downstream and antisense to *Dusp9* and does not disrupt polyadenylation or splicing of either gene. Knock-in mice engineered to carry the ETn display *Ppd* characteristic ectopic caudal limb phenotypes, showing that the ETn insertion is the *Ppd* molecular lesion. Early transposons are actively expressed in the early blastocyst. To explore the consequences of the ETn on the genomic landscape at an early stage of development, we compared interval gene expression between wild-type and mutant ES cells. Mutant ES cell expression analysis revealed marked upregulation of *Dusp9* mRNA and protein expression. Evaluation of the 5′ LTR CpG methylation state in adult mice revealed no correlation with the occurrence or severity of *Ppd* phenotypes at birth. Thus, the broad range of phenotypes observed in this mutant is secondary to a novel intergenic ETn insertion whose effects include dysregulation of nearby interval gene expression at early stages of development.

## Author Summary

Mobile genetic elements, particularly early transposons (ETn), cause malformations by inserting within genes leading to disruption of exons, splicing or polyadenylation. Few mutagenic early transposon insertions have been found outside genes and the effects of such insertions on surrounding gene regulation is poorly understood. We discovered a novel intergenic ETnII-β insertion in the mouse mutant *Polypodia (Ppd)*. We reproduced the mutant phenotype after engineering the mutation in wild-type cells with homologous recombination, proving that this early transposon insertion is *Ppd*. Mutant mice are not born in expected Mendelian ratios secondary to loss after E9.5. Embryonic stem cells from mutant mice show upregulated transcription of an adjacent gene, *Dusp9*. Thus, at an early and critical stage of development, dysregulated gene transcription is one consequence of the insertion mutation. DNA methylation of the ETn 5′ LTR is not correlated with phenotypic outcome in mutant mice. *Polypodia* is an example of an intergenic mobile element insertion in mice causing dramatic morphogenetic defects and fetal death.

## Introduction

The molecular causes of vertebrate malformations and the molecular basis of the variability in Mendelian syndromes are incompletely understood. While coding alterations have received a substantial amount of attention, the contribution of variation or mutation in intergenic regions, as well as the role of genetic background/modifiers, epigenetic and environmental factors, retrotransposons and transgenerational genetic effects, are receiving more attention particularly in relation to penetrance, expressivity and pleiotropy [1]–[8].

Spontaneous mobile element insertions in mice can be associated with alterations in body plan and morphogenesis [9]. There are many types of transposable elements; however, those active in the mouse are mostly IAP or Type II early transposons (ETn) [9]. Type II early transposons carry long terminal repeats (LTR) and are classified into MusD, ETnI and ETnII subtypes. IAP, MusD and ETnII insertions are responsible for a substantial fraction (∼10%) of spontaneous new mutations in mice [9]. Most previously reported mutagenic ETn insertions occur in the sense orientation within genes, resulting in disruption of exons, polyadenylation and/or splicing. ETn elements are highly transcribed during pre-gastrulation and at later stages of morphogenesis in selected tissues [10–12] and while promoter activation of adjacent genes has been demonstrated for IAP elements, it has not been observed for ETn insertions [9]. Moreover, ETn regulatory sequences such as enhancers and repressors upon random insertion in new genomic environments could exert deleterious or beneficial effects on neighboring gene expression. The activity of retrotransposons varies depending on their state of methylation, which is controlled by host factors, and many transposable elements act as metastable epialleles [9,13,14].

**Figure 1:**
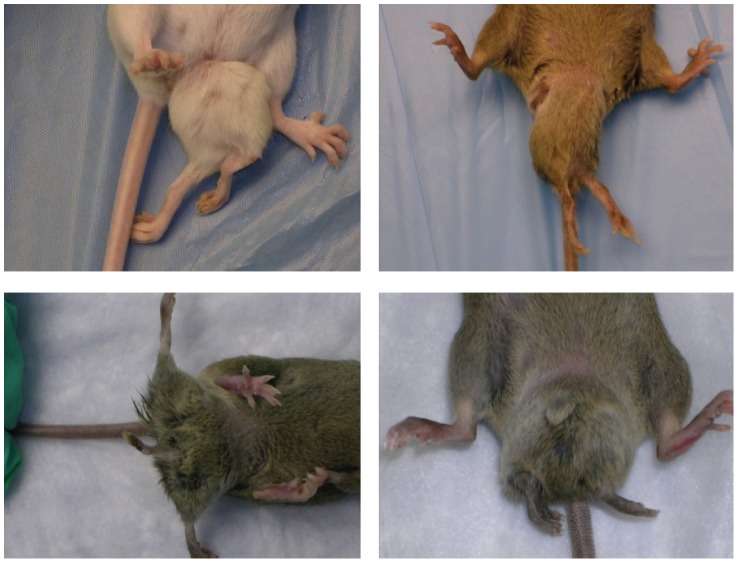
Typical *Ppd* caudal duplications, among numerous anomalies observed in mutants. Please see [15] for a complete description of other anomalies, listed in the Introduction. Variation in presentation of ectopic legs with or without caudal masses has been observed.

Previously we reported the phenotypes and genetic mapping of *Polypodia*, (*Ppd*), a dominant, X-linked mouse mutation exhibiting malformations in 20–25% of newborn mutation carriers [15]. Postnatally affected mice predominantly exhibit ventral, caudal limb duplications (Figure 1) and a variety of other defects including bilaterally asymmetric anomalies, partially duplicated snouts and whiskers, mirror-image pelvic duplication (dipygus), extra digit-like bony growths on abdominal skin, cystic kidneys, renal agenesis, duplicated external genitalia with normal internal genitalia, kinked, curly or knotted tails, forelimb postaxial polydactyly, radial aplasia, spina bifida, microphthalmia (unilateral), supernumerary nipples, yet no malignancy, duplicated upper extremities, or extra spinal elements. We localized the mutation to a ∼10 Mb interval on the mouse X-chromosome between markers *DXMIT74* and *rs13483835* [15]. The striking body plan alterations offer an opportunity to understand in molecular terms how such disorganization of the vertebrate body plan can occur and how these principles might inform our understanding of similar birth defects in humans.

In this paper, we 1) show that *Ppd* mutant embryos are not born at expected Mendelian ratios due to fetal loss, 2) describe the discovery of a novel, intergenic ETnII-β insertion in the refined genetic interval, 3) recreate the mutation using homologous recombination in ES cells and recapitulate *Ppd* phenotypes, 4) show that one effect of the *Ppd* ETn insertion is dysregulated adjacent gene transcription in mutant ES cells, and 5) show that the state of DNA methylation of the 5′ LTR is not correlated with *Ppd* phenotypic variability.

## Results

### Mutant mice are not born in expected Mendelian ratios secondary to loss after E9.5

*Ppd* arose on the CD-1 strain and mutants exhibit a variety of malformations as described above, although the ventral, caudal duplications with extra limbs are the most frequent and dramatic [15]; Figure 1. We crossed *Ppd* hemizygous males and heterozygous females to the wild-type, inbred C3H/HeJ strain for over 10 generations and observed that ∼21% of mice born with *Ppd* interval genetic markers [15] showed abnormal phenotypes. We attempted crosses to produce a higher frequency of postnatal anomalies to facilitate later experimental studies by outcrossing *Ppd* mice (male or female) on the C3H background (generation N8) to CAST/EiJ, CZECHII/EiJ, MSM/Ms, C3H/HeJ, C57BL/6J, DBA/2J, CD1, and B6/D2 F_1_ hybrids. Offspring were evaluated at birth for any of the phenotypes observed in *Ppd* mutants and genomic DNA was collected and genotyped for the *Ppd* haplotype [15]. In this breeding scheme, inclusion of C57BL/6J genetic background did not change the frequency of observed postnatal malformations (∼21–22%) in females or males bearing the *Ppd* genetic interval. Outcrossing for one generation to CAST/EiJ, CZECHII/EiJ and DBA/2J chromosomes resulted in the lowest percentage with birth anomalies (∼0–0.4%), whereas ∼11–14% of newborns of MSM/Ms, B6/D2 and CD1 outcrosses had anomalies at birth. This is not a formal measure of penetrance. It suggests, but does not prove, that genetic background could have a significant effect on the phenotypic outcome related to inheriting this mutation, but evidence to support that conclusion will require many generations on the individual strains as well as examination of both prenatal and postnatal phenotypes.

We hypothesized that apparent variations in the frequency of postnatal malformations in mutants at birth might be influenced by embryonic lethality. To test this, we took advantage of a genetic cross for mapping purposes that produced *Ppd* heterozygous female mice with one wild-type CZECHII X-chromosome and one *Ppd* X chromosome (C3H background) and mated these females with wild-type C3H males. Offspring of this latter cross were genotyped for interval markers and sex as described [15], which allowed us to determine the birth frequency of male and female offspring with the *Ppd* chromosome, which must come from the female. Table 1 shows the X-chromosome identity in offspring (CZECHII/C3H refers to a female with CZECHII and C3H chromosomes; CZECHII/Y refers to a male with a CZECHII X-chromosome; *Ppd*/C3H refers to a female with *Ppd* and C3H X-chromosomes; *Ppd*/Y refers to a male with a *Ppd* X-chromosome). A 60% reduction of the *Ppd* haplotype was found in liveborn males and a 23% reduction was observed in liveborn females (Fisher's Exact test, p<0.007). A similar result was obtained in a cross involving only the C3H background (82% and 36% reductions, respectively; Table 2; p<0.055). The data indicate that there are fewer *Ppd* mutants at birth than expected and males with *Ppd* are more likely than females to fail to be born.

**Table 1:** Reduced *Ppd* chromosomes at birth – CZECHII/EiJ cross.

X chromosomes	Females	Males
CZECHII/C3H or CZECHII/Y	148	122
*Ppd*/C3H or *Ppd*/Y	114	54

**Table 2:** Reduced *Ppd* chromosomes at birth – C3H cross.

X chromosomes	Females	Males
C3H/C3H or C3H/Y	25	22
*Ppd*/C3H or *Ppd*/Y	16	4

To determine if *Ppd* X-chromosomes are represented in offspring early in development as expected, we evaluated the genotypes and sex of conceptuses at E9.5. *Ppd* males (C3H background) were crossed to CD-1 females, followed by a backcross of female *Ppd* offspring to CD-1 wild-type males. Evaluation of those offspring revealed expected numbers of *Ppd* X-chromosomes in conceptuses at E9.5 (Table 3). Thus, embryos must be dying between E9.5 and birth. Our preliminary data suggest that mutants occasionally display extensive early gastrulation abnormalities including overallocation of extraembryonic tissue at the expense of the epiblast and accumulation or piling up of cells in the primitive streak (J. Innis, K. Downs, P. Wakenight, K. Millen, data not shown). Further work will be required to determine the basis of fetal loss in these mutants.

**Table 3:** Expected Mendelian numbers of *Ppd* X-chromosomes at E9.5.

	Female	Female	Male	Male	
**Gestational age**	*Ppd* hets	WT	*Ppd*	WT	Total
**E9.5**	24	24	22	24	94

### The *Ppd* genetic interval harbors a novel ETnII-β insertion

We reported the location of *Ppd* in a 9.64 Mb genetic interval on the X- chromosome [15]. To narrow the interval, we crossed our *Ppd* mice on the C3H background to CZECHII/EiJ mice to exploit a greater number of polymorphic differences and improve crossover resolution. Using 2 visibly affected recombinant animals, we narrowed the interval to 1.85 Mb between DXMIT94 and rs13483824.a at 72.02 Mb and 73.87 Mb, respectively (GRCm38). In addition, we test crossed the visibly unaffected critical recombinant F_2_ animals and looked for affected progeny, allowing us to refine our map based on the *Ppd* “carrier” haplotype. These efforts allowed us to locate *Ppd* in a ∼1.4 Mb interval between DXMIT119 and SNP rs13483824.a (data not shown).

**Figure 2:**
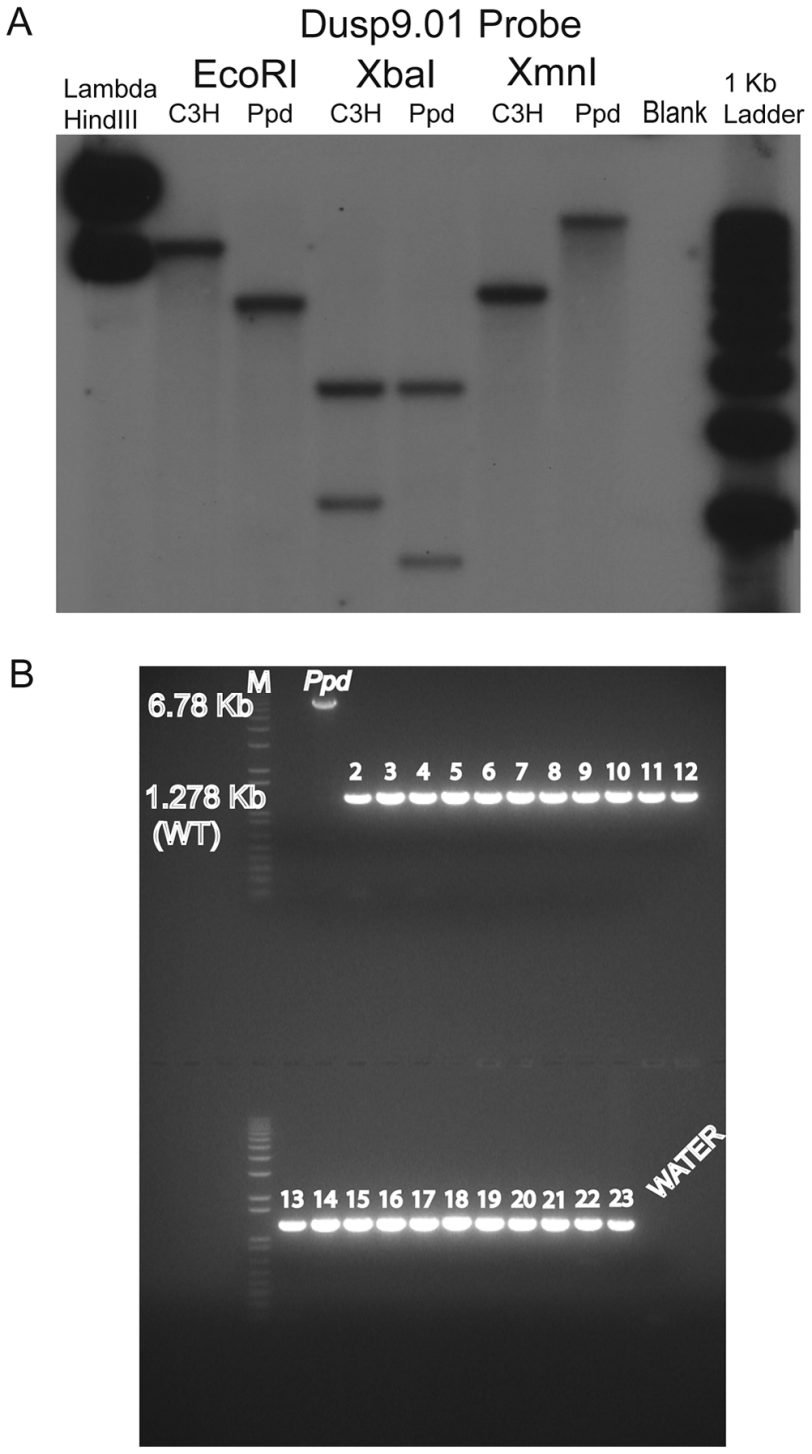
A novel DNA insertion mutation within the *Ppd* genetic interval. A. Southern blot demonstrating abnormal product sizes in male *Ppd* genomic DNA compared to male C3H DNA hybridized with Dusp9.01 probe. Southern blot comparisons with other strains revealed these alterations to be *Ppd*-specific (Figure S1). B. The *Ppd* insertion is not present in 21 different mouse strains and CD-1 (original strain). X-chromosome interval-specific PCR primers (F1/R2, see Figure 3) were used to amplify affected male *Ppd* DNA compared with male genomic DNA samples (obtained independently from Jackson Labs) from the following strains: Lane 2: 129S1/SvImJ; 3: 129X1/SvJ; 4: A/J; 5: AKR/J; 6: Balb/cByJ; 7: Balb/cJ; 8: C3H/HeJ; 9: C57BL/6J; 10: C57BL/10J; 11: CAST/EiJ; 12: CBA/J; 13: CD10/JlsJ; 14: CZECHII/EiJ; 15: DBA/1J; 16: DBA/2J; 17: FVB/NJ; 18: MOLF/EiJ; 19: MSM/Ms; 20: SJL/J; 21: SPRET/EiJ; 22: SWR/J. Representative outbred CD-1 (Charles River Labs) genomic DNA is shown in lane 23 adjacent to the water control. Analysis of 11 other independent male CD-1 mouse DNA samples also revealed only the 1.28 kb wild-type PCR product (not shown). The 6.8 kb product spanning the insertion was only observed using *Ppd* DNA; the 1.28 kb wild-type product is labeled.

**Figure 3:**
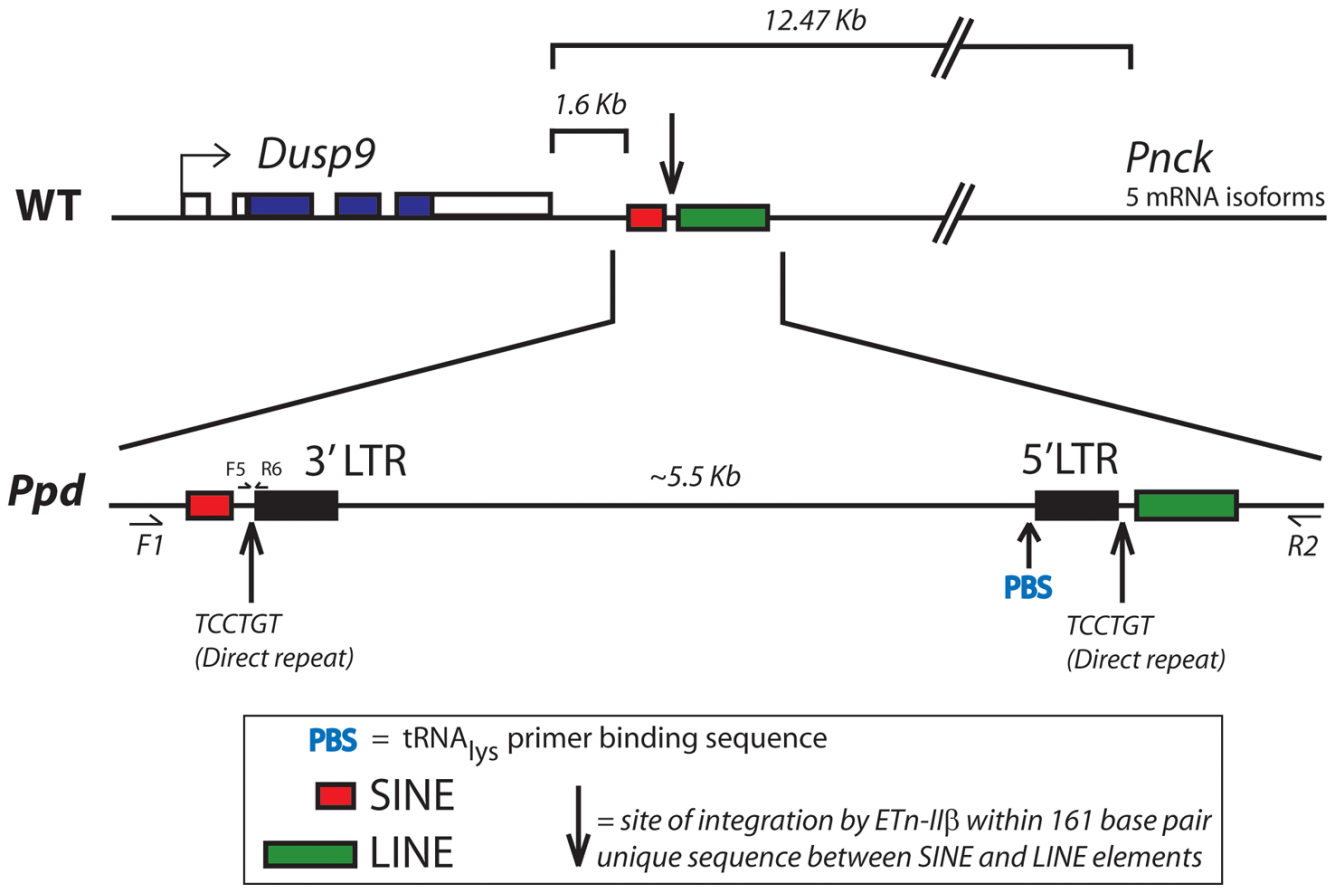
Location and orientation of new ETnII-β insertion in the *Ppd* genetic interval relative to immediately adjacent genes. The ETn insertion site is located between *Dusp9* and *Pnck*, ∼1.6 kb downstream and antisense to *Dusp9*. Transcription proceeds from the 5′ LTR toward *Dusp9*. PCR primers used for genotyping mutants (F5/R6) are shown.

We previously reported a normal karyotype and no apparent submicroscopic gene dosage aberration by BAC array comparative genomic hybridization (CGH) [15]. To examine the X chromosome in more detail, we compared male *Ppd* DNA to wild-type male C3H DNA using an X-chromosome-specific NimbleGen array in a CGH experiment with average probe spacing every 500 base pairs. No variation was identified on the X-chromosome within the 1.4 Mb critical genetic interval (data not shown). Thus, at this level of resolution *Ppd* is not due to a chromosomal deletion/duplication, leaving us to consider single gene smaller mutations, deletions or insertions. Our refined genetic mapping experiments on the X-chromosome defined a *Ppd* interval with over 30 annotated protein coding genes. To determine if *Ppd* was a mutation in one of these interval genes, we prioritized gene candidates based on known gene function and initiated a variant search with several methods. Southern analysis with non-repetitive, gene-centered DNA probes and *Ppd* genomic DNA disclosed altered restriction digest patterns with a *Dusp9* gene probe (Figure 2A). This alteration was not observed with this probe in other mouse strains (Figure S1). Using PCR primer walking and DNA sequencing of PCR products and clones spanning the entire insertion and flanking regions we identified a 5.5 kb insertion positioned 1.6 kb downstream of the 3′ end of the *Dusp9* gene (Figure 3). No mutations of endogenous chromosomal material were observed in adjacent genomic regions. We demonstrated absence of this genomic alteration in representative background (CD-1) male genomic DNA, as well as 21 different mouse strains using PCR (Figure 2B). Similarly affected mutant mice were independently discovered by K. Millen and P. Wakenight in CD-1 animals at the University of Chicago. Blinded testing with a *Ppd* mutation-specific PCR assay utilizing unique primers to the adjacent X chromosome and the newly inserted sequences (see Figure 3, primers F5/R6; 248 base pair product), demonstrated the same insertion mutation in those affected mice (data not shown).

The DNA sequence of the inserted segment (GenBank Accession: Mouse_ETnII-B_Polypodia_X_Chromosome_DNA KC512757) revealed it to be an early transposon type IIβ (ETnII-β) element. This conclusion is supported by 1) the sequences of the homologous 5′ and 3′ LTRs; 2) the presence of a putative Lys-tRNA binding site (PBS) 5′-TGGCGCCCGAACAGGGA-3′, 3) the presence of a 6 bp direct duplication (5′-TCCTGT-3′ in the orientation shown in Figure 3) at the insertion junctions, 4) absence of coding sequences that would be more characteristic of MusD or IAP elements [16–18], 5) absence of ETnI-specific sequences [19], and 6) the presence of specific sequences found only in ETnII-β elements that cross an internal deletion (ETnII-3636as = 5′-GTCACTTAATACCCCCTGACTAACAAATG-3′; [20,21]. The *Ppd* interval ETnII-β is highly related to several endogenous ETnII-β elements located on chromosome 5 (AC163331), chromosome 13 (AC163684) and within the desmoglein locus, among others. As expected, the 317 bp LTRs of the newly identified ETn are identical and have 16 CpG dinucleotide sites.

The *Ppd* interval ETn is located 1.6 kb downstream (relative to *Dusp9* transcription) of the polyadenylation signal of *Dusp9*, between two repetitive sequences (SINE and LINE elements; Figure 3) at position ChrX: 73645160 (GRCm38/mm10). This insertion does not disrupt *Dusp9*, *Pnck*, or any other known gene or noncoding RNA; examination of the EST databases shows no reported spliced or unspliced ESTs or isoforms beyond exon 4 of *Dusp9* or of the last exon of *Pnck*. Sequencing of exons and exon/intron boundaries of *Dusp9* and *Pnck* did not reveal any pathogenic sequence variants. The orientation of the ETn is antisense to *Dusp9* gene transcription and the insertion site is located ∼10.8 kb from the 3′ end of the *Pnck* gene. Thus, the ETn insertion appeared to be a strong candidate for *Ppd*. While transposon insertions are well known mutagens, the intergenic position of the insertion was novel.

### Insertion of the *Ppd* ETn into the wild-type genome reproduces *Ppd* phenotypes

**Figure 4:**
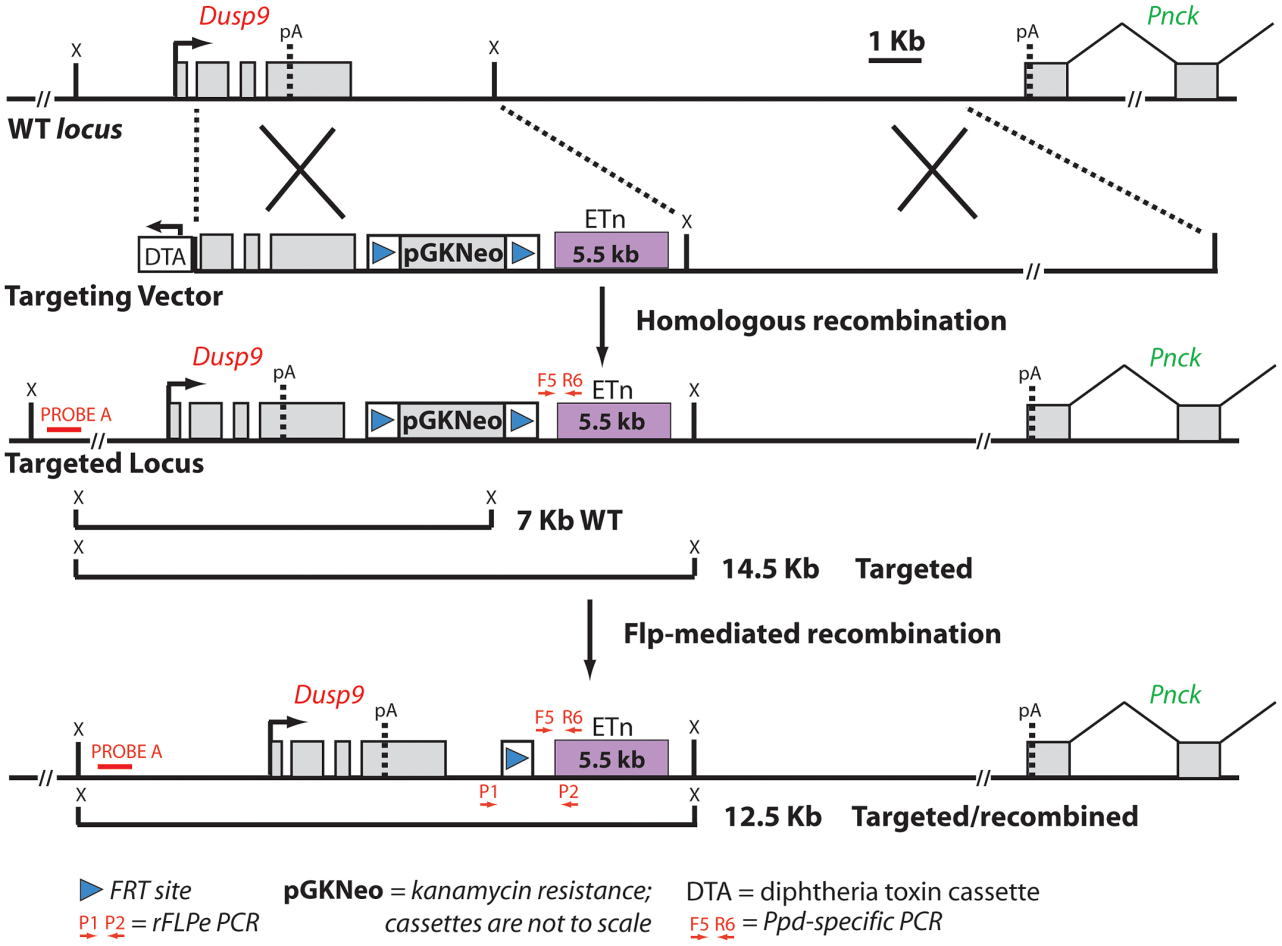
Schematic diagram of the targeting vector strategy and expected results of homologous recombination and Flp-mediated selection cassette removal. Ppd BAC clone 2 was the starting material from which a 5′ homology segment of 5 kb beginning in *Dusp9* intron 1 at GRCm38 position ChrX: 73639873 including exons 2, 3, and 4, as well as a 3′ homology arm extending 10 kb toward *Pnck* including the ETn and ending at GRCm38 position ChrX: 73655955, was excised from the BAC and used for recombineering as described in Materials and Methods. ES cell clone DNA was subjected to Southern blotting with Probe A (located outside of the 5′ homology arm) after XmnI digestion (labeled as X restriction site in the figure; see also Figure S2), as well as PCR with F5 and R6, to identify successful homologous recombinants. FLP-mediated recombination between the two FRT sites (blue triangles) and removal of the PGK-Neo cassette was accomplished by mating Neo^+^/eETn^+^ females with β-actin FLPe male mice obtained from Jackson Labs. Successful FLP-mediated recombination was verified in offspring by PCR/sequencing with X chromosome specific primer P_1_ (5′-CAAATGCCTGAGCTGATAAAATAA-3′) and LTR specific primer P_2_ (5′-CCCTTCCTTCATAACTGGTGTC-3′) (see Figure S3).

**Figure 5:**
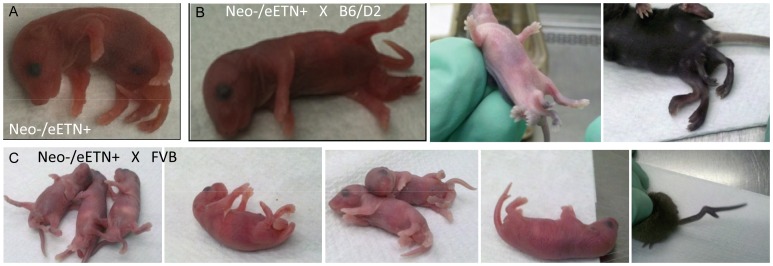
*Ppd* characteristic caudal mass/ectopic limb phenotypes in engineered offspring. A. (one panel) Bruce4.G9 targeted ES cell clone (9281)-derived chimeric male produced a female heterozygous carrier (Neo^+^/eETn^+^) that was mated to a β-actin FLPe male to produce the newborn (Neo^−^/eETn^+^) shown in the panel here. Genotyping confirmed the proper FLPe recombination fragment demonstrating Neo cassette removal in this mouse. B., C. Typical caudal masses with ectopic limbs in offspring of Neo^−^/eETn^+^ mice. 9 out of 69 offspring from mating between Neo^−^/eETn^+^ males X B6/D2 F_1_ females demonstrated typical caudal masses/ectopic limbs (3 panels in B), whereas 8 out of 31 offspring from mating between Neo^−^/eETn^+^ males X FVB females demonstrated typical caudal masses and in one mouse a bifurcated tail seen at birth and later at 3 weeks (5 panels in C).

To determine whether this novel intergenic ETnII-β insertion is *Ppd*, we sought to introduce this ETn into a wild-type genome to create an engineered ETn allele (eETN). We first created a BAC library from male *Ppd* genomic DNA and then isolated a BAC clone spanning the genomic region including the ETn. We used BAC recombineering to construct a targeting vector for homologous recombination in mouse ES cells (Figure 4). DNA sequencing of 5′ and 3′ genomic targeting arms was employed to determine whether the ETn insertion was the only plausible candidate mutation in the targeting vector. Sequencing disclosed one common, non-coding SNP variant (rs29038663; C>T; GRCm38/mm10) by comparison with the reference C57BL/6J sequence. Thus, the ETn insertion is the only candidate mutation within the targeting vector. We employed Bruce-4.G9 (a chromosomally stable sub-line generated at the University of Michigan Transgenic Animal Core Lab from Bruce4 ES cells) [22] and UMB6J-D7 (a pure BL/6 line generated here at the University of Michigan) mouse ES cell lines to knock-in the ETn into the wild-type genome. Three hundred clones from each electroporation were picked and expanded. Southern blotting with Probe A (see Figure 4) and *Ppd* ETn-specific locus PCR (F5/R6) confirmed a high frequency of homologous recombination in both cell lines (27–50%). Five ES cell clones from each line were karyotyped and 5 cell lines (4 Bruce4.G9 and 1 UMB6J-D7) from those clones were found to be euploid. All euploid lines were reexamined by Southern blotting (Figure S2) and by *Ppd*-specific PCR (not shown) and were found to be correctly targeted. Blastocysts were injected with the Bruce-4.G9 targeted ES cells, and chimeric males were produced. Germline transmission was successful in generating 10 female engineered ETn (eETn) heterozygotes (Neo^+^/eETn^+^); none of these females exhibited an abnormal phenotype. We bred these females to β-actin FLPe males (Jackson Lab stock #005703), to excise the Neo cassette and demonstrated expected PCR products after excision (Figure S3). Figure 5A shows a Neo^−^/eETn^+^ progeny female with a caudal mass and ectopic legs. This observation confirmed our hypothesis that the ETn is the *Ppd* mutation. To determine if phenotypically unaffected Neo^−^/eETn^+^ mice could have offspring with *Ppd* phenotypes consistent with the original *Ppd* mutant, we bred Neo^−^/eETn^+^ carrier males to B6/D2 F_1_ hybrid or FVB females. Nine out of 69 (13%) eETN^+^ offspring of B6/D2 mothers and 8 out of 31 (26%) eETN^+^ offspring of FVB mothers, had caudal masses with ectopic limbs. These results demonstrate that germline transmission of the engineered allele from the male or female germline is associated with typical *Ppd* caudal malformations (Figure 5B, C). Moreover, in this small cohort on mixed genetic backgrounds, the frequency of postnatal malformations and phenotypic variability in the engineered lines is similar to that of the original *Ppd* allele. These results confirm that the ETnII-β insertion is the *Ppd* mutation.

### *Ppd* mutant ES cell lines overexpress *Dusp9* mRNA and protein

Endogenous retroviral transpositions including ETnII-β insertions are the cause of ∼10% of spontaneous new mouse mutants [9,19]. Most, but not all, mutagenic ETn insertions occur within genes in the mouse and are sense-oriented [9,23]. Transcriptional interference with splicing or 3′ end formation, when ETn insertion occurs within genes due to the contribution of ETn splice sites and polyadenylation signals, is well documented and is the basis of most phenotypic effects of such insertions [9]. To begin to explore the mechanism by which the *Ppd* ETn insertion was interfering with development, we first examined the structure and expression of flanking genes *Dusp9* and *Pnck* mRNAs in mutant embryos.

*Dusp9* encodes a MAP kinase tyrosine/serine/threonine phosphatase of which there are numerous family members [24,25]. *Dusp9* is expressed in ES cells [26], but it is not essential for ES cell viability, although BMP4 has recently been shown to activate *Dusp9* transcription via SMAD1/5, resulting in reduction of pERK in ES cells [27]. Expression also has been observed in the ectoplacental cone and chorion of the placenta as early as E7; at E8.5 *Dusp9* is activated in the ventral foregut endoderm, which ultimately becomes the liver. It is also expressed in dorsal and ventral muscle groups of the forelimb and hindlimb at E9–E11; the face (E9), mandible and hypoglossal cord [24]. *Dusp9* heterozygous and null mutants die prenatally by E10.5 due to failure of growth of the placental labyrinth [26], and by tetraploid rescue mutants exhibit normal embryonic development [26]. *Pnck* encodes a pregnancy-upregulated, non-ubiquitously expressed calcium/calmodulin-dependent serine/threonine protein kinase [28], and is known to be expressed in mammary glands, brain and during hippocampal dendritic growth. PNCK has also been shown to induce ligand-independent epidermal growth factor receptor degradation [29]. Therefore, we sought to test if the ETn alters *Dusp9* or *Pnck* 3′ RNA structure by evaluating mRNA from E7–E9.5 whole mutant embryos compared to wild-type littermates by 3′ RACE. No major differences were detected in relative abundance or in 3′ RACE products of *Dusp9* or *Pnck* RNA in mutant embryos at these developmental times (Figure S4).

We hypothesized that the ETn may ectopically activate or interfere with the transcription of *Dusp9* or *Pnck*, through modification of the chromatin environment or through enhancer provision, usage, or interference. This hypothesis seemed particularly relevant considering the burst of early transposon transcription that occurs during early stages of development from E3.5–E7.5 [10–12,30]. To test this hypothesis, we first examined the mRNA expression and structure of *Dusp9* and *Pnck* in wild-type mouse embryonic stem cells. ES cells represent the inner cell mass at a developmental stage when early transposon transcription is high. Reverse-transcription PCR using oligo-dT primed synthesis followed by PCR using primers in different exons confirmed that *Dusp9* and *Pnck* are normally expressed in wild-type ES cells (data not shown). Due to the close location of the ETn to *Dusp9*, we used mutant ES cells to evaluate *Dusp9* splicing (from exons 2–4 by RT-PCR) and 3′ end formation as assessed by 3′ RACE. Neither were disrupted in mutant ES cells (data not shown), consistent with the observations in mutant embryos.

**Figure 6:**
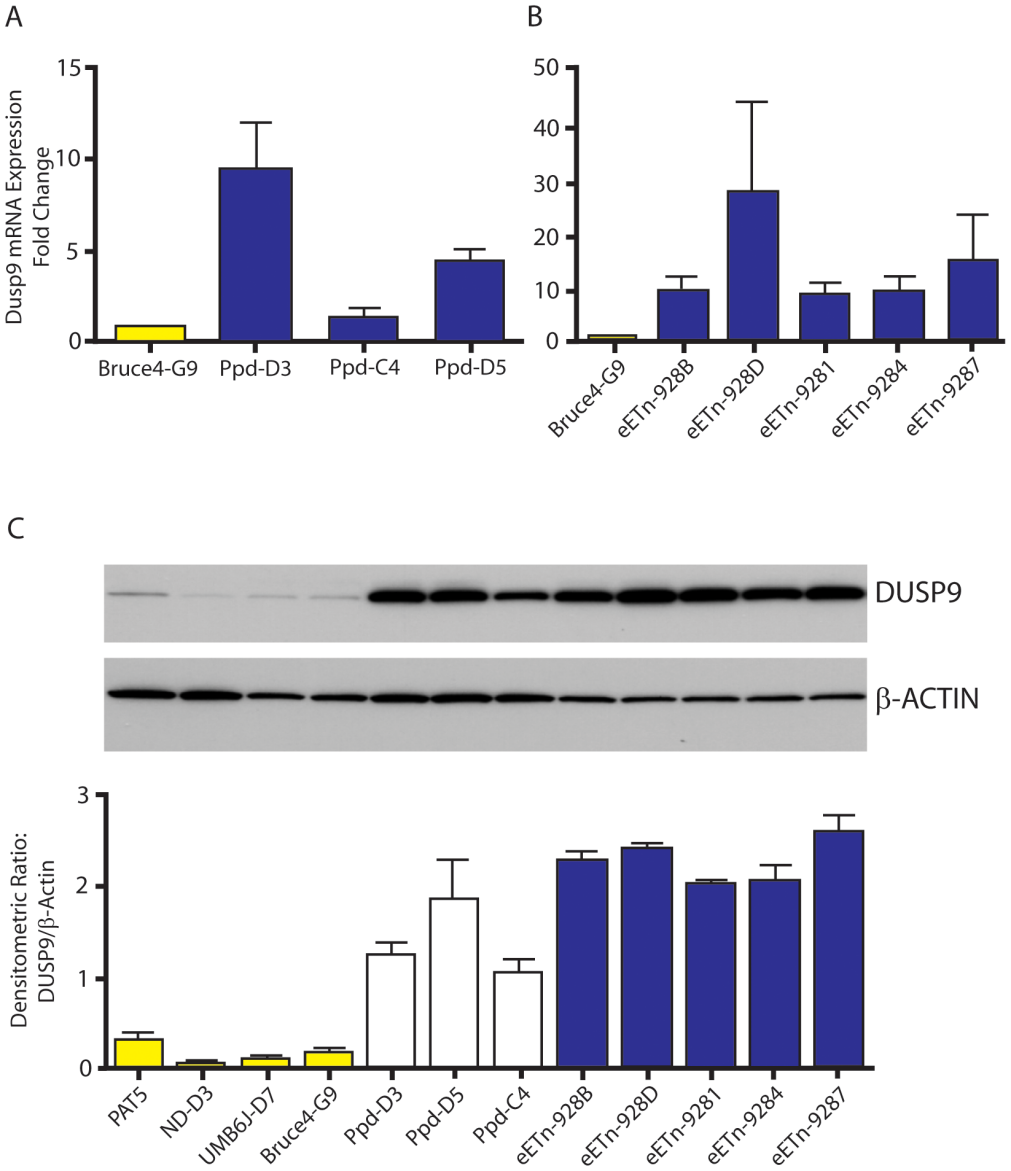
*Dusp9* mRNA and protein are increased in mutant ES cells. A, B. Taqman confirmation of increased *Dusp9* mRNA in mutant ES cells. ES cell steady-state *Dusp9* mRNA quantitation in ES cell lines (D3, C4, and D5) derived from the original *Ppd* strain (see Materials and Methods) compared with Bruce4.G9 ES cell RNA. Steady-state *Dusp9* mRNA levels in engineered eETn targeted ES cell lines (928B, 928D, 9281, 9284, 9287) derived by homologous recombination in Bruce4.G9 cells, compared to *Dusp9* expression in wild-type Bruce4 ES cells. All targeted ES cell lines also demonstrate a large increase in *Dusp9* mRNA compared to Bruce4.G9; n = 3 independent RNA preparations. Note variability of RNA expression across different ES cell lines. C. Increased expression of DUSP9 protein in mutant ES cells. DUSP9 protein was quantitated by Western blot with antibody to DUSP9 (top blot), produced and kindly supplied by R. Dickinson, relative to β-actin (lower blot) from normal ES cells (PAT-5, ND-D3, UMB6J-D7, and Bruce4.G9) compared to original *Ppd* mutant-derived ES cells (Ppd-D3, Ppd-D5, Ppd-C4) and eETn ES cells (as shown on right, blue bars); graph below represents data from two separate protein extraction experiments. Bruce4.G9 is the parent ES cell line for eETn cells. Data with the second antibody (anti-MKP4; Santa Cruz Biotech.) was identical (data not shown). Pre-incubation of each antibody with a synthesized peptide to DUSP9 reduced or eliminated the signal observed in ES cells, and pre-incubation with a control peptide did not visibly alter the DUSP9 signals (Figure S5).

To determine if *Dusp9*, *Pnck* or other X chromosome local interval gene transcription is dysregulated as a consequence of the ETn insertion, we examined steady-state mRNA from several independent mutant male ES cell lines using Affymetrix Mouse GeneChip 430 2.0 expression microarrays. We compared all 3 original *Ppd* ES lines with normal ES cell mRNA prepared from Bruce4.G9, ND-D3 and UMB6J-D7 lines. We focused our analysis to genes in 500 kb intervals on either side of the ETn insertion site on the mouse X chromosome. Within this 1 Mb interval are 35 RefSeq genes (GRCm38/mm10), for which 9 were not represented on the microarray used (2 microRNA genes; 4 X-linked lymphocyte regulated genes; and 3 newly added genes in mm10, *Haus7*, *Naa10* and *Tex28* not located close to the ETn insertion site). Both *Dusp9* and *Pnck* were represented. Genes in this interval whose expression fulfilled quality measures (see Materials and Methods), were increased or decreased at least 2 fold and exhibited a FDR≤0.05, were *Dusp9* (all 3 probe sets, increased 3.12, 2.74 and 2.6 fold) and *Slc6a8* (only 1 of 2 probe sets, increased 2.34 fold and 1.07 fold). *Pnck* mRNA expression was not altered. *Slc6a8*, which encodes a brain creatine transporter, is located telomeric to *Pnck* and was not examined further. We used Taqman real-time quantitative RT-PCR directed to *Dusp9*, a MAP kinase phosphatase, to confirm the array result. Steady-state *Dusp9* RNA expression was elevated in all ETn-bearing ES cells by 5–15 fold over wild-type cells (Figure 6A, B). To determine if the elevated levels of *Dusp9* steady-state mRNA are associated with higher levels of steady-state DUSP9 protein, we performed Western blots with protein extracts from mutant ES cell populations compared to 4 different wild-type ES cell lines (Figure 6C). Western blots with DUSP9 antibody (gift from Robin Dickinson; [24]) revealed increased DUSP9 protein expression (7–14 fold), adjusted for β-actin, in all *Ppd* ES cell lines and all eETn ES cell lines. This was confirmed with an independent antibody (data not shown). The specificity of both antibodies for DUSP9 was confirmed by testing the effects of pre-incubation with synthesized DUSP9 peptide versus control, nonspecific peptide (Figure S5). Thus, DUSP9 protein is over-expressed in *Ppd* mutant ES cells. We conclude that one consequence of ETn insertion is *Dusp9* overexpression in pluripotent cellular representatives of the inner cell mass.

### Variable occurrence of postnatal *Ppd* phenotypes is not explained by variation in *Ppd* ETn 5′ LTR methylation

Retrotransposon activity varies depending on the state of methylation of the locus [13,14]. CpG methylation increases from 5′ to 3′ within individual ETn LTRs [14]. We hypothesized that variable occurrence in the *Ppd* phenotype among ETn carriers or *Ppd* males at birth may be explained by variation in *Ppd* interval ETnII-β 5′ LTR methylation. To test this hypothesis, we used bisulfite sequencing of tail genomic DNA from affected versus unaffected *Ppd* ETn carrier (female) and male littermates. After bisulfite modification, we amplified 237 base pairs of the 317 bp 5′ LTR anchoring on adjacent X-chromosome specific genomic sequence, allowing us to interrogate seven 5′ LTR-specific CpG dinucleotides and 1 adjacent X chromosome genomic CpG dinucleotide immediately upstream of the transcription start sites mapped in ETnII-β elements [21]. Comparison showed that inter-individual differences in the occurrence of a *Ppd* phenotype at birth is not related to the methylation state of the 7 CpG dinucleotides in the 5′ portion of the 5′ LTR (Table 4) in either females or males. We also examined the methylation of the ETn in *Ppd* ES cells; the ETn, as expected, was largely unmethylated at this stage of development. In addition, male *Ppd* animals, regardless of phenotype, exhibited a broader distribution of the degree of methylation of these 8 CpGs. To determine if variation in 5′ LTR methylation was observed between tissues within an affected animal, genomic DNA derived from normal tissue (tail) and from the caudal ectopic legs/mass from one adult *Ppd* female was subjected to bisulfite sequencing. No differences were observed in the degree or distribution of methylated CpG residues. These results suggest that if the methylation state of the ETn does affect the occurrence of postnatal phenotypes, it is not observable as a difference in 5′ LTR methylation in adult tissues.

**Table 4:** Variation in occurrence of malformations at birth is not correlated with 5′ LTR promoter methylation.

	Methylated-CpGs	8	7	6	5	4	3	2	1	0	Sequenced Clones
**NL-** ***Ppd*** ** carriers**											
#3318		10	17	3	2	1	1	2	0	0	36
#3413		12	16	2	1	1	0	0	0	0	32
**A-** ***Ppd*** ** carriers**											
#3317		7	3	3	1	1	1	0	0	0	16
#3412		14	15	6	3	0	0	0	0	0	38
#3412(cm)		15	8	13	2	0	0	1	0	0	39
**NL ** ***Ppd*** ** male**											
#3567		3	4	4	5	5	7	3	5	0	36
**A-** ***Ppd*** ** male**											
#3568		6	5	4	7	9	7	5	1	0	44
**Ppd-D5 ES cells**		0	0	0	0	0	0	1	5	30	36

Independent clones generated by PCR were sequenced and analyzed for CpG methylation at 8 sites (7 LTR and 1 X chromosome genomic) upstream of the 5′ LTR in 2 *Ppd* carrier females with normal phenotypes (NL-Ppd) versus 2 *Ppd* carrier females with abnormal phenotypes (A-Ppd). The number of clones for each sample with the indicated number of methylated CpGs are tabulated. No difference was observed at these CpG sites either in number or distribution of sites of methylation between these groups. For comparison, CpG methylation was also assessed in affected and unaffected *Ppd* males and in ES cell line Ppd-D5. In this ES cell line the LTR is almost completely unmethylated, whereas in postnatal adult males the ETn LTR is methylated to a variable extent, but less than in females. cm = caudal mass tissue of mouse #3412.

## Discussion

Using genetic mapping and homologous recombination in ES cells, we have shown that a novel ETnII-β insertion discovered to lie 1.6 kb downstream of the *Dusp9* gene is the *Ppd* genetic lesion. ETnII-β elements often insert into exons and disrupt splicing and polyadenylation [9], yet we find no evidence of an altered *Dusp9* transcript structure. Instead, in mutant ES cells, one apparent effect of the ETn in this new genomic environment is increased *Dusp9* mRNA and protein expression. ES cells represent the pluripotent inner cell mass at a developmental time point associated with increased ETn transcription and it is attractive to speculate that interference, by an as yet unknown mechanism, with appropriate transcriptional regulation of *Dusp9* at this or other stages of development, or of other genes in this region of the X chromosome, gives rise to the phenotypic effects in the *Ppd* mutant. ETn elements have been hypothesized to exert mutational effects on gene expression at a distance, but few examples have been identified. *Dactylaplasia* [31] is due to MusD (ancestral ETn) element insertion within (Dac2J) or upstream (Dac1J) of the *dactylin* gene [32], and the two mutant alleles are suppressed by an unlinked modifier, *Mdac* [31]. Limb defects in *Dactylaplasia* mice may result from *Mdac*-suppressible transcriptional interference with apical ectodermal ridge expression of *Fgf8* [32–34], a gene located more than 70 kb away from the MusD insertion sites. Interestingly, MusD expression in the AER is increased in mutant limbs suggesting that *Fgf8* AER enhancers may be co-opted by an active MusD element in this mutant [34]. In addition, *Mdac* appears to dominantly modulate the MusD methylation state, which inversely correlates with the phenotype. Recently, another intergenic ETn insertion 12.5 kb upstream of *Ptf1a* was elucidated as the cause of the semidominant Danforth's short tail (*Sd*) mutation, and this insertion is associated with upregulation of embryonic expression of *Ptf1a* leading to caudal regression phenotypes [35–37]. The addition of our example confirms that such intergenic insertions, while rare, are capable of modifying gene expression, although in all cases reported so far, the mechanism remains to be determined.

In contrast to *Dac* mutants, the methylation state of the *Ppd* 5′ LTR is not correlated to phenotype. These results are consistent with prior conclusions indicating that ETnII transcriptional activity is regulated by more than methylation state and genomic environment [21]. Although we did not examine the 3′ LTR, which is closest to the *Dusp9* gene, histone modification and chromatin structure across the *Dusp9*/ETn interval could be altered by the ETn and would be exciting to examine in future studies, with consideration given to analysis of selected cell populations earlier in development.

We have not proven that upregulation of *Dusp9* or modification of any other interval gene expression is the cause of the malformations and/or fetal death. It is conceivable that ETn transcriptional effects (negative or positive) could also occur at later developmental phases in different tissues. ETn expression occurs in two phases [10–12]. In the first phase, ETnII transcription occurs during E3.5–E7.5 beginning in the inner cell mass and extending into the epiblast and extraembryonic ectoderm. The 2^nd^ phase occurs between E8.5–E11.5 beginning with E8.5 neural tube ETnII expression outlining the rhombomeres [12]. This neural expression gradually decreases as mesodermal expression increases in the somites at E8.5. At E9.5–10.5, expression is observed in the olfactory placode and then becomes concentrated along the nasal pit and lateral nasal processes. Strong branchial arch ETnII expression was observed at E8.5–E11.5. Finally, the forelimb and hindlimb buds exhibited strong expression at E9.5 and E10.5, respectively. At E11.5, ETnII expression was noted in the condensing ulna/radius. Since there are 300–400 copies of type II ETn/MusD elements in the mouse genome, expression analyses likely reflect the contribution of expression from multiple genomic locations. Interestingly, this multiphasic, multiple tissue expression pattern could, in part, be related to the varied organ effects of the ETn insertion in *Ppd* mutant mice. For example, the ETn could ectopically activate *Dusp9* in ES cells in association with the early burst of ETn transcription normally observed at E3.5. In this situation, proximity to *Dusp9* creates an opportunity for *Dusp9* dysregulation consequent to the insertion of a transcriptionally activated ETn nearby. Potential interference with *Dusp9* or other interval genes in specific tissues at later times is a natural hypothesis to examine as the etiology for malformations. It is intriguing that normal *Dusp9* expression occurs later in development in other regions of the embryo as described [12,24] (including the olfactory placode and nasal pit, somites and limbs) that overlaps tissue malformations observed in some *Ppd* mutants: double snouts, spina bifida, and ulnar aplasia, syndactyly or hypodactyly [15].

*Ppd* mice strikingly resemble the mouse mutants *Disorganization* [38–41] and *Duplicitas posterior* [42–44], as well as conceptuses exposed to the teratogen all-*trans* retinoic acid (RA) at pre-gastrulation stages, E4.5–E5.5 [45–48]; [Innis et al., unpublished]. Ducks, cows, deer and other animals have also been reported (not shown) with similar *Ppd*-like, dramatic caudal or other ectopic limb duplications, suggesting that common fundamental vertebrate developmental pathways are susceptible to spontaneous mutations or environmental teratogens. Humans with ectopic lower limbs with and without pelvic anomalies or dipygus, have been described extensively in the literature [49–56]; all cases occurred sporadically, not unlike the occurrence of *Ppd*.

*Duplicitas posterior* mice had varying pelvic masses and accessory limbs identical to *Ppd* mutants [42–44]. This mutation, which was never identified, arose on the stock carrying *Sd*, Danforth's short tail, had a penetrance of 20% in liveborn mice, caused prenatal death in some, and showed significant strain variation in penetrance and phenotype. Embryologically, Danforth noticed a thickening at mouse gestational age E11 of the “ventral tissues at the posterior end of the embryo in a region including, and extending in front of, the usual site of the cloacal pit”. The cloaca was noticed to widen out laterally and form two cloacal membranes, often resulting in two urethrae. Generally the mice had only 1 rectum, but occasionally two were observed, as might be expected from cloacal thickening. Duplicated pelvic bones, kinked tails, agenesis or hypoplastic kidneys (suggesting interference with mesonephric duct development), microphthalmia and other anomalies were noted. These are quite similar to the defects we described for *Polypodia* mice [15]. Danforth also identified some mutants with double spinal cord at the lumbar/thoracic region and variations in between, as well as neural tube defects. Subsequent studies found a duplicate neural tube without notochord in some E11–E12.5 mutant pelvic masses suggesting bifurcation or budding off from the primary neural tube secondary to duplication of organizer tissue or the primitive streak, but this was not formally examined [44]. We have not observed duplicated neural tubes in *Ppd* mutants, although we have seen split tails and some spinal dysraphism on a few occasions on the genetic backgrounds presented. Unfortunately, *Duplicitas posterior* mice no longer exist (E. Center, personal communication).

The mouse mutant *Disorganization* (*Ds*) causes a wide variety of malformations in the mouse compatible with an early postimplantation patterning disruption. This mutation maps to mouse chromosome 14. *Ds* mice share many malformations [38–41] in common with those of *Polypodia*, yet there are differences. *Ds* mice do not exhibit prenatal lethality, either as heterozygotes or homozygotes [40]. It will be interesting to compare the molecular pathways affected in both mutants.

Exogenous retinoic acid (RA), given at E4.5–E5.5 (blastocyst stage), produces a mouse *Ppd* phenocopy. Such mouse conceptuses develop caudal limb and lower body duplications [45–48]; [Innis et al., unpublished], duplicated genital buds, facial defects and exencephaly. RA-treated embryos also display facial anomalies, which were not described in detail [45], although these were more frequently observed when RA exposure occurred on E6–E7. In most affected embryos, normal hindlimb development, single tails, and ectopic, ventral, rudimentary or complete lower limbs or caudal structures with or without duplicated pelvic structures are produced. The susceptible gestational times (E4.5–5.5) correspond to post-implantation stages before gastrulation. Thus, provision of RA at E4.5–5.5 to pregnant dams clearly reorganizes the mouse body plan, and since RA is cleared within 12 hours of administration [57,58] the effect of RA is immediately confined to cells at pre-gastrulation stages. We believe that *Ppd*, *Ds*, and retinoic acid exposure at E4/5–E5.5 impact similar developmental pathways leading to caudal duplications and other malformations.

Sporadic mutants for which coding alterations are elusive may be secondary to similar spontaneous insertions. However, it remains to be determined how *Ppd* and these other models intersect within known developmental pathways and at what developmental timepoint(s). Moreover, the principles that influence penetrance, expressivity and pleiotropy in *Ppd* phenotypes are certainly relevant to human disease.

## Materials and Methods

### Ethics statement

All mouse experiments were approved by the UM University Committee on the Use and Care of Animals, Protocol #07982.

### Mouse husbandry and genetic crosses

Genetic crosses were carried out as described [15]. For narrowing the *Ppd* genetic interval, we genotyped visibly affected recombinant animals and utilized extended crosses (offspring exceeding 80–100 animals for each) of visibly unaffected CzechII/C3H F_2_ critical recombinants.

### Southern blotting

Non-repetitive mouse genomic DNA segments were amplified by PCR and sequence verified to use as probes in Southern blots with ten micrograms of restriction enzyme digested mouse genomic DNA from wild-type and *Ppd* mutant mice. A 2212 bp *Dusp9* probe, DUSP9.01, corresponding to GRCm38 genomic coordinates ChrX:73641114–73643326 that includes *Dusp9* gene sequences from the middle of intron 2 through most of the 3′ UTR of exon 4, was amplified with primers 5′-GGGCACTTATCAGCCAAAGA-3′ and 5′-GGTGTGGACTGCAATGAATG-3′. This DNA segment was labeled with ^32^P-dCTP and used according to standard Southern hybridization and washing protocols. ES cell genomic Southern blots were carried out as described [59].

### *Ppd* ETn genotyping

X-chromosome specific primers used to amplify across the *Ppd* ETn as shown in Figure 2B were F1 (5′-AGCAAATGGTGGGACTGTGTAAT-3′) and R2 (5′-ACCCAGGACGATTGAAGATGTGC-3′), which together generate a 1.278 kb product on wild-type DNA, but a 6.778 kb product including the ETn. Tail genomic DNA for genotyping was isolated by overnight proteinase K digestion, followed by extraction with phenol/chloroform/isoamyl alcohol and ethanol precipitation. *Ppd* mutation-specific PCR was performed using F5 (X-chromosome specific) and R6 (ETn LTR) primers that yielded a 248 bp *Ppd*-specific product in mutants. PCR success was assessed by including wild type forward and reverse primers in the same PCR that yielded a wild type product of 100 bp. Male *Ppd* mutant PCR yields only the 248 bp *Ppd* -specific product.

F5 – 5′-TTACCAGGAGAAAGGACGCACTATGAG-3′

R6 – 5′-GCACCTTTCTACTGGACCAGAGATT-3′

WT Forward – 5′-TTGGGTCAAAGTTGAATGAAAATAGAAATAGC-3′

WT Reverse – 5′-CCCCGCCACTTCAGTGCTACC-3′

Thermocycling was carried out in 25 µL, 0.5 M betaine and 3 mM MgCl_2_ with an initial 2-min 97°C denaturation followed by 36 cycles of 97°C for 30 sec, 63°C for 30 sec and 72°C for 30 sec. The final extension was for 5 min at 72°C.

### Quantitative RT-PCR

Real-time RT-PCR was performed on an ABI Prism 7000 thermocycler (Applied Biosystems, Foster City, CA. Gene-specific primers and probes were designed using Primer 3 program. Sequences for primers and probes for mouse *Dusp9*, *Pnck* and *β-actin* are as follows:

Mouse *β-actin* Forward Primer –AAGAGCTATGAGCTGCCTGA

*β-actin* Reverse Primer – CAAGAAGGAAGGCTGGAAAAGAG

Probe – 6FAMAACGAGCGGTTCCGATGCCCTGTAMRA

Mouse *Dusp9* Forward Primer – GGCATCCGCTATATCCTCAA

*Dusp9* Reverse Primer – GGGGATCTGCTTGTAGTGGA

Probe – 6FAMCCCCAACCTTCCTAACCTCTTAMRA

Mouse *Pnck* Forward Primer – CTCCCGGTTTTTCTTTCCTC

*Pnck* Reverse Primer – ATGCATCACACCCAGTCTCA

Probe – 6FAMTGGATCCTTGTCCTCCAGACTAMRA

RNA was extracted using TRIzol reagent (Invitrogen) from at least three independent preparations of mouse ES cells, Ppd-ES cells and eETn ES cells. Each RNA sample (0.5 µg) was tested in triplicate using TaqMan one-step RT-PCR master mix reagents from Applied Biosystems. Average cycle threshold (C_T_) was determined for each sample and normalized to β-actin. Relative gene expression (using the formula 2^−ΔΔCT^ ) was calculated using the comparative CT method, which assesses the difference in gene expression between the gene of interest (*Dusp9*) and an internal standard gene (*β-actin*) for each sample to generate the ΔCT [59]. The difference of the ΔCT for each experimental cell line from the ΔCT the control cell line BRUCE4.G9 is referred to as ΔΔCT. The average of the control sample (BRUCE4.G9) was set to 1 for each experiment, and the relative gene expression (fold change) for each experimental sample was compared with that.

### Creation of *Ppd* ES cells

We obtained *Ppd* blastocysts by mating 24–28 day old pseudopregnant *Ppd* CD-1 (>90% CD-1) females, recovering blastocysts at E3.5 by uterine flushing, and single-well plating on feeder cells. Following the identification of male cells carrying the *Ppd* ETn, we established mutant ES cell lines Ppd-D3, Ppd-D5, and Ppd-C4.

### Mouse ES cell culture

ES culture procedures were performed as described in [60]. Mouse ES cells were maintained on γ-irradiated mouse embryonic fibroblasts (PTMN cells: pretreated, mouse embryonic fibroblasts, neomycin resistant) in Dulbecco's modified Eagle's medium (DMEM) supplemented with 15% fetal calf serum (Atlanta Biologicals), 1000 U/ml LIF (Millipore), 4 mM L-glutamine, 1% non-essential amino acids, 0.1 mM β-mercaptoethanol, 1% sodium pyruvate, and 1% penicillin/streptomycin. For RNA/DNA/protein analysis, ES cells were grown on gelatin coated plates without feeder cells, passed twice sequentially to eliminate PTMN feeder cell contamination, in DMEM with 15% fetal calf serum and 1000 U/ml LIF.

### Mouse ES cell RNA extraction and Affymetrix gene expression analysis

RNA was isolated using TRIzol from ES cells after passage twice sequentially on gelatin coated plates without feeder cells. Biotinylated cDNA was prepared from 50 ng total RNA according to the Nugen ovation V2 kit protocol (NuGen, Inc.). Following labeling, 4 µg of cDNA was hybridized for 16 hours at 45°C on GeneChip Mouse 430 2.0 arrays. GeneChips were washed and stained in the Affymetrix Fluidics Station 450 and then scanned with an Affymetrix 3000 7G GeneChip Scanner. Data quality analysis revealed no degradation and robust *in vitro* translation. Standard error estimates for each gene were derived and then standardized across all arrays, all of which showed high quality samples. A robust multi-array average (RMA) modeling strategy [61] was used to convert the PM probe values into expression values for each gene. Since we compared three normal ES cells lines to three *Ppd* ES cell lines, we used weighted linear models [62], pooling information from all probe sets, to stabilize the variance estimate. Weighting was accomplished by a gene-by-gene algorithm that downweights samples deemed less reproducible [63]. We removed probe sets across sample comparisons (Male WT versus Male *Ppd*) that had a variance of less than 0.1 and then selected genes with a fold-change greater than 2 and an adjusted *p*-value (adjusted for multiple comparisons using false discovery rate, FDR) of less than 0.05 [64]. We used the Affy, AffyPLM and limma packages of Bioconductor in the R statistical environment.

### Homologous recombination

To place the ETn into a wild-type mouse genome, we first created a BAC library (in vector pIndigoBAC5) from *Ppd* male genomic DNA utilizing the services of Bio S&T (Lachine, Quebec) and isolated 2 BAC clones spanning the *Ppd* ETnII insertion site on the X chromosome and surrounding genes spanning over 170 kb. We selected one clone (Ppd BAC Clone 2) with a 170 kb insert and used BAC recombineering to construct a targeting vector through the UC Davis Mouse Biology Program (http://mouse.ucdavis.edu/ineed/vectors_constructs.php). The strategy of construction began with the BAC. *Ppd* BAC Clone 2 was electroporated into EL350 and selection with chloramphenicol was used to isolate colonies. A frt-flanked PGK-Neo was inserted into the BAC just upstream of the 5.5 kb ETn insert via BAC recombineering and clones were selected with kanamycin (PGK-Neo confers kanamycin resistance in bacterial cells), and chloramphenicol. The region containing the ETn, frted PGK-Neo, and 5′ (5 kb) and 3′ (10 kb) arms of homology was retrieved into a high-copy plasmid followed by selection with kanamycin and ampicillin (retrieval vector confers Amp^r^). A Gateway reaction was then used to swap in the DTA negative selection marker followed by selection with kanamycin, which replaced the retrieval vector portion, and removed the Amp^r^ cassette. Finally, a separate electroporation to isolate the targeting vector with the insertion followed by kanamycin selection was performed. Sequencing of all junctions created by recombineering revealed the expected insert structure. Sequencing of the 5′ (5 kb) and 3′ (10 kb) endogenous mouse genomic DNA arms of the targeting vector revealed not only the ETn, but also one common non-coding SNP, rs29038663, a C>T substitution at ChrX:73646920, 1,767 base pairs telomeric (closer to the *Pnck* gene) to the ETn. We targeted Bruce-4.G9 and UMB6J-D7 (a pure BL/6 line) ES cell lines. Three hundred ES cell clones from each electroporation were picked and expanded. Southern blotting and *Ppd* ETn-specific locus PCR revealed a very high frequency of homologous recombination in both cell lines (27–50%). Germline transmission was successful in generating female engineered ETn (eETn) heterozygotes (Neo^+^/eETn^+^). We bred these females to β-actin FLPe males (Jackson Lab stock #005703), to remove the Neo cassette and obtained germline Neo^−^/eETn^+^ mutant mice for phenotypic analysis.

### RNA isolation from mouse embryos (E7.5) and genotyping

*Ppd*-CD-1 mutant female mice were kept for overnight mating with a CD-1 WT male. Conception was defined by the presence of a vaginal plug the following morning, and the age of embryos calculated from midnight. Pregnant *Ppd*-CD-1 female mice were euthanized by carbon dioxide asphyxiation at E7.5. Embryos were immediately dissected from the uterus in cold PBS under a dissecting microscope, and a portion of the ectoplacental cone and yolk sac were used for DNA isolation. Briefly, 20 µL alkaline lysis reagent (25 mM NaOH/2 mM EDTA) was added to the tissue samples, and the mixture was incubated at 95°C for 20 minutes followed by neutralization using 20 µL 40 mM Tris-HCl. Genomic DNA was then used for genotyping using sex and *Ppd* genotyping. RNA was extracted from the embryos using Trizol reagent (Invitrogen) according to the manufacturer's instructions. Embryo sex was determined as described [15] using XX-XY forward and reverse primers that produce a ∼300 bp single product in females and a doublet in males. Thermocycling was carried out in 25 µL containing 0.5 M betaine and 3 mM MgCl2 with an initial 2-min 97°C denaturation followed by 36 cycles of 97°C for 30 sec, 63°C for 30 sec and 72°C for 30 sec. The final extension was for 5 min at 72°C. Primers: XX-XY forward: CCGCTGCCAAATTCTTTGG; XX-XY reverse: TGAAGCTTTTGGCTTTGAG. *Ppd* genotyping was as described above.

### Protein extraction and Western blotting

ES cells grown on tissue culture plates were washed with phosphate-buffered saline (PBS) and lysed in 0.4 ml of ice-cold RIPA lysis buffer (1% sodium deoxycholate, 0.1% SDS, 0.15 M NaCl, 0.01 M NaH_2_PO_4_, 2 mM EDTA, 0.5 mM NaF) containing 2 mM sodium orthovanadate and 1∶1000 dilution of protease inhibitor mixture III (Calbiochem). Protein concentrations were determined using the DC protein assay reagents from Bio-Rad (Hercules, CA). SDS-PAGE and Western blot analysis were performed. Cell lysates were mixed with a 1∶5 v/v ratio of 6× gel loading dye (0.35 M Tris-HCl pH 6.8, 30% glycerol, 10% SDS, 0.6 M DTT, 0.012% bromophenol blue) and boiled at 95°C for 5 min to denature proteins. Sample mixtures were then loaded on 4–20% polyacrylamide gradient gels and subjected to electrophoresis. Proteins were electrophoretically transferred to a polyvinylidene difluoride membrane (Immobilon–P, Millipore Inc., Bedford, MA) and incubated in 1× Tris-buffered saline (pH 7.4), 0.1% Tween 20 with 5% bovine serum albumin for 1 h at room temperature. The blot was incubated with 1∶1000 dilution of primary antibody in blocking buffer overnight at 4°C. Three washes with 1× TBS with 0.1% Tween 20 were performed prior to incubation with a secondary antibody conjugated to horseradish peroxidase. The washes were repeated five times, and the membrane was incubated with SuperSignal West Pico chemiluminescent substrate (Thermo Scientific, Rockford, IL) for 5 min. The blot was then exposed to chemiluminescent-sensitive HyBlot CL autoradiography film (Denville Scientific Inc., Metuchen, NJ). Image analysis was performed using a public domain NIH Image program available on the internet at rsb.info.nih.gov/nih-image.

### Antibodies and peptides

Sources of antibodies used in this study were as follows. Sheep anti-mouse DUSP9 polyclonal antibody, raised against two DUSP9 peptides (residues 237–261 and residues 429–451; [24]) was a gift from Dr. Robin Dickinson, University of Dundee, UK. From Santa Cruz Biotechnology (Santa Cruz, CA): MKP-4 rabbit polyclonal antibody raised against a single DUSP9 peptide corresponding to residues 231–270. From Bio-Rad: HRP conjugated anti-sheep secondary antibody. From Thermo Scientific (Rockford, IL): Peroxidase conjugated goat anti-rabbit IgG and peroxidase conjugated anti-mouse IgG. Mouse monoclonal β-actin antibody was from Sigma. Synthesized peptides (DUSP9 peptide 231–274 and a PNCK 30 amino acid peptide) used in specificity assays were produced in the UM Protein Structure Facility.

### Bisulfite sequencing

Tail samples were taken from 14 day old mice. Genomic DNA from an adult animal was used for comparison of LTR methylation between tail or other organ versus caudal ectopic mass. DNA was prepared from the samples and PCR was performed to confirm the presence of the ETn insertion. Once confirmed, the DNA was purified and treated with bisulfite using established protocols in the Qiagen EpiTect Bisulfite Kit. The bisulfite treated DNA (btDNA) samples were subjected to PCR using the primers EpiF4 (5′- GGTAAAAGAAGAAATGTAGTTAAGATAGTT-3′) targeting the modified LTR, and EpiR5 (5′- AAACTCCCCAAAACAAAACACTATA -3′) targeting the modified X chromosome sequences (ChrX:73645196–73645220) upstream of the 5′ LTR. One reaction contained, 15.6 µL ddH_2_O, 2.5 µL 10× JumpStart PCR Buffer, 0.5 µL dNTP's, 1.25 µL Primer F4, 1.25 µL Primer R5, 0.4 µL JumpStart Taq, and 2.5 µL of 5 M Betaine. Each reaction also contained ∼200 ng of btDNA. The PCR program used was: 97°C (2 min), 97°C (30 sec), 46°C (30 sec), 72°C (1 min), Step 2 (40×), 72°C (10 min), 4°C (∞). A second round of PCR was set up identical to the first, except 2 µL from the first round of PCR was used as the template for the second round PCR. No purification was necessary between PCR rounds. PCR reaction products were separated by electrophoresis on a 2% agarose gel. The bands were extracted and purified using a Qiagen Gel Extraction Kit. The PCR products were TA-cloned into a pGEM-T easy vector. The ligation was then electroporated into DH5α cells and plated onto LB agar with carbenicillin. Individual colonies were selected and grown overnight. Plasmid DNA from individual colonies was extracted and individual clones were sequenced in the University of Michigan DNA Sequencing Core with T7 and SP6 primers. Bidirectional sequences were scanned for the targeted CpG dinucleotide as well as unmethylated cytosine modifications.

## Supporting Information

Figure S1 *Ppd*-specific restriction fragments identified in Southern analysis with a Dusp9.01 probe. Genomic DNA from *Ppd* and various mouse strain samples was digested with XbaI or EcoR1, subjected to electrophoresis and blotted to nylon membranes. Each was hybridized with the Dusp9.01 DNA probe. *Ppd*-specific bands (red arrows) are not observed with other strain DNA samples. *Ppd*-m is a male mouse; *Ppd*-f416 is a heterozygous mutant female.
(TIF)

Figure S2 Southern analysis of targeted ES cell clones. ES cell genomic DNA was purified from clones 9281, 9283, 9284, 9285 and 9287, several normal mice and one *Ppd* mouse and digested with XmnI. The DNA was separated by electrophoresis, blotted and hybridized with labeled DNA Probe A (Figure 4). All ES cell lines shown demonstrate the expected 14.5 kb XmnI fragment expected for correct targeting. Clone 9281 was used to inject blastocysts to generate chimeric males.
(TIF)

Figure S3 Demonstration of recombination by FLPe in offspring of Neo+/ETn+ mice mated to β-actin FLPe mice by PCR. Genomic DNA was isolated from offspring and subjected to PCR with primers P1 and P2 as described. The expected PCR product size was identified in all offspring and DNA sequencing (not shown) disclosed the expected sequence.
(TIF)

Figure S4 3′ RACE of *Dusp9* and *Pnck* mRNA expression reveals normal polyadenylation. RNA and genomic DNA were isolated from E7.5, E8.5, and E9.5 WT & *Ppd* mutants using TRIzol (Invitrogen). Genomic DNA was used for *Ppd* and sex genotyping [15] of each embryo. One µg of total RNA from each embryo was used for reverse transcription using Superscript III (Invitrogen) and PCR primers F6 (*Dusp9* exon 4) or F2B (PNCK last exon) and Inv-3′RACE Invitrogen primer. No differences were detected in the 3′ ends of RNA in mutant embryos. Identical assays with ES cell transcripts were normal (not shown). “?” refers to failure to determine genotype as either WT or *Ppd*.
(TIF)

Figure S5 DUSP9 antibodies are specific to DUSP9. ES cell protein extracts were separated by electrophoresis and subjected to Western blotting with DUSP9 antibody (R. Dickinson) or commercially available antibody from Santa Cruz Biotech against MKP-4. To test for specificity, each antibody was pre-incubated with synthesized DUSP9 peptide as described in Materials and Methods. Pre-incubation of DUSP9 antibodies with a specific peptide against one of the epitopes used to make the R. Dickinson antibody and the entire epitope used to make the MKP-4 antibody, reduces or eliminates Western blot signal; whereas, pre-incubation with a nonspecific PNCK antibody did not affect either signal.
(TIF)
