# Supplementary figures and images for: A Novel Intergenic ETnII-β Insertion Mutation Causes Multiple Malformations in Polypodia Mice
Source: PLoS Genet. 2013 Dec 5;9(12):e1003967. doi: 10.1371/journal.pgen.1003967 (PMC3854779; doi:10.1371/journal.pgen.1003967)

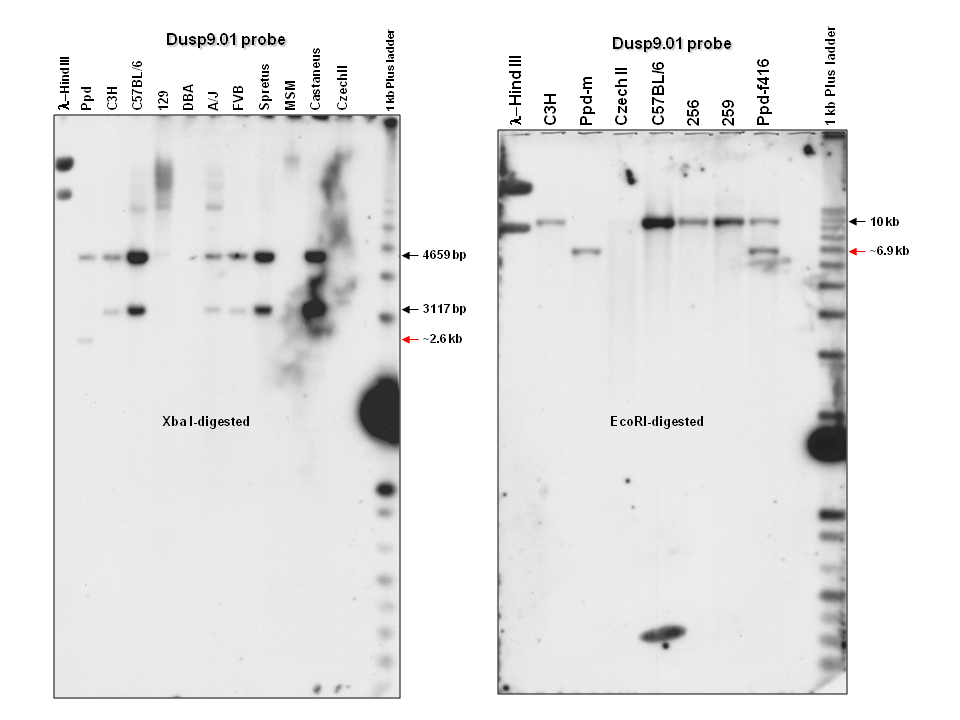

Supplement: Figure S1 — Ppd-specific restriction fragments identified in Southern analysis with a Dusp9.01 probe. Genomic DNA from Ppd and various mouse strain samples was digested with XbaI or EcoR1, subjected to electrophoresis and blotted to nylon membranes. Each was hybridized with the Dusp9.01 DNA probe. Ppd-specific bands (red arrows) are not observed with other strain DNA samples. Ppd-m is a male mouse; Ppd-f416 is a heterozygous mutant female. (TIF) [file pgen.1003967.s001.tif]

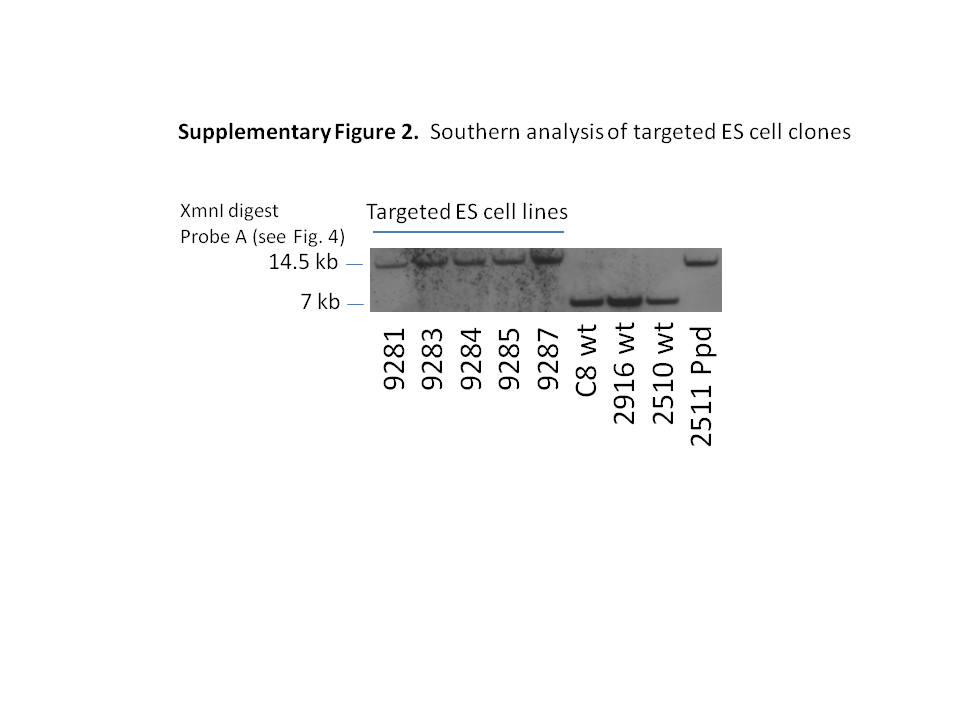

Supplement: Figure S2 — Southern analysis of targeted ES cell clones. ES cell genomic DNA was purified from clones 9281, 9283, 9284, 9285 and 9287, several normal mice and one Ppd mouse and digested with XmnI. The DNA was separated by electrophoresis, blotted and hybridized with labeled DNA Probe A (Figure 4). All ES cell lines shown demonstrate the expected 14.5 kb XmnI fragment expected for correct targeting. Clone 9281 was used to inject blastocysts to generate chimeric males. (TIF) [file pgen.1003967.s002.tif]

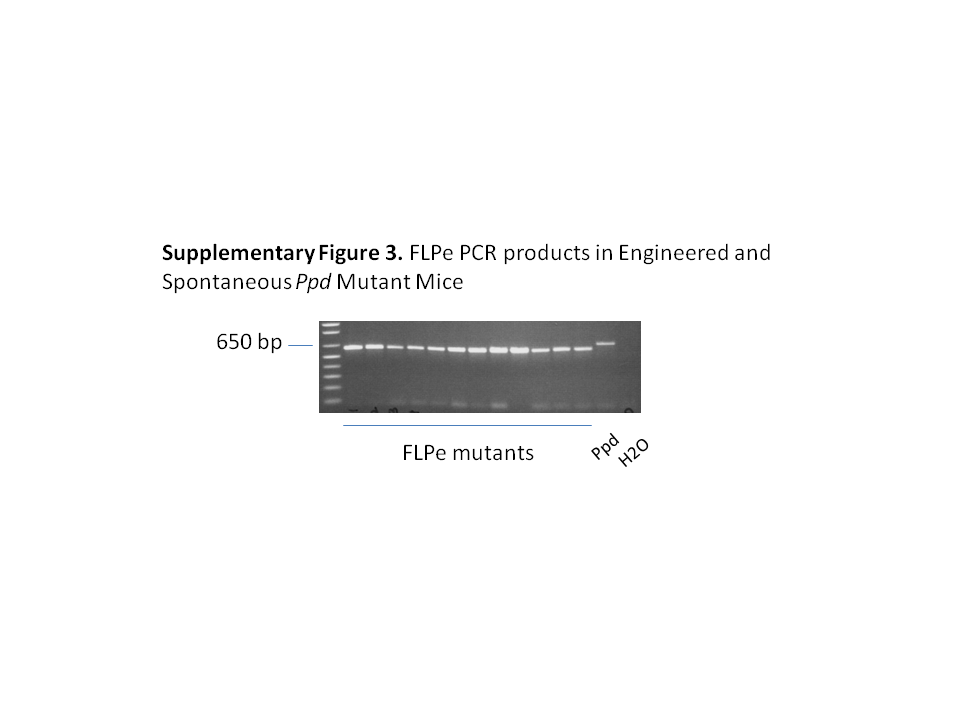

Supplement: Figure S3 — Demonstration of recombination by FLPe in offspring of Neo+/ETn+ mice mated to β-actin FLPe mice by PCR. Genomic DNA was isolated from offspring and subjected to PCR with primers P1 and P2 as described. The expected PCR product size was identified in all offspring and DNA sequencing (not shown) disclosed the expected sequence. (TIF) [file pgen.1003967.s003.tif]

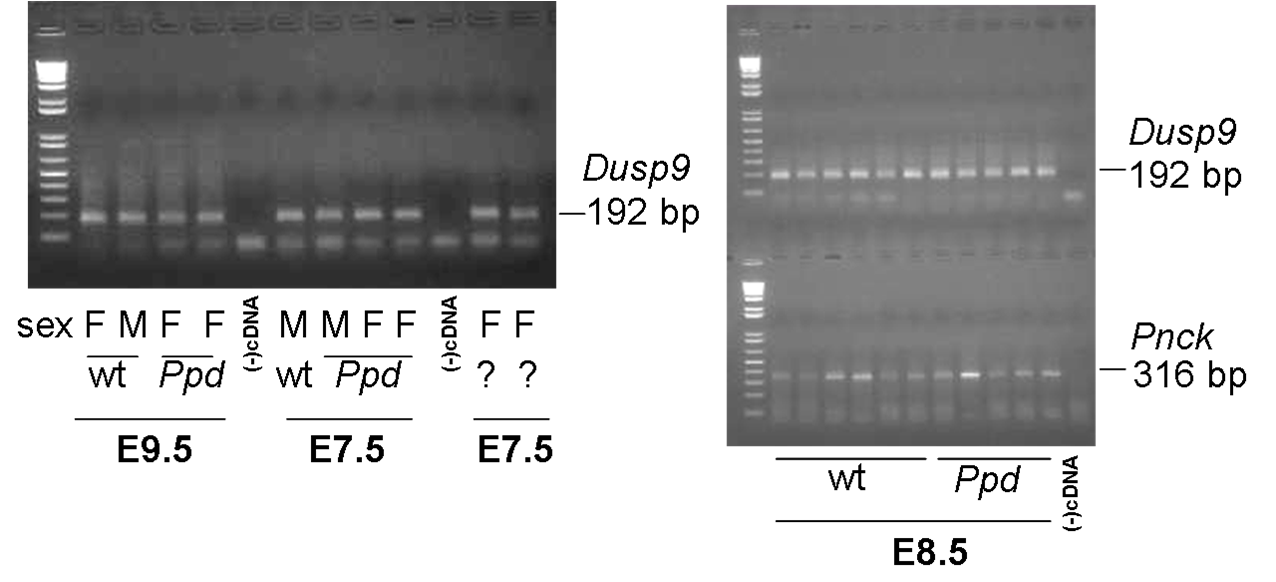

Supplement: Figure S4 — 3′ RACE of Dusp9 and Pnck mRNA expression reveals normal polyadenylation. RNA and genomic DNA were isolated from E7.5, E8.5, and E9.5 WT & Ppd mutants using TRIzol (Invitrogen). Genomic DNA was used for Ppd and sex genotyping [15] of each embryo. One µg of total RNA from each embryo was used for reverse transcription using Superscript III (Invitrogen) and PCR primers F6 (Dusp9 exon 4) or F2B (PNCK last exon) and Inv-3′RACE Invitrogen primer. No differences were detected in the 3′ ends of RNA in mutant embryos. Identical assays with ES cell transcripts were normal (not shown). “?” refers to failure to determine genotype as either WT or Ppd. (TIF) [file pgen.1003967.s004.tif]
